# First comparative analysis of complete chloroplast genomes among six *Hedysarum* (Fabaceae) species

**DOI:** 10.3389/fpls.2023.1211247

**Published:** 2023-08-18

**Authors:** Inom Juramurodov, Dilmurod Makhmudjanov, Ziyoviddin Yusupov, Komiljon Tojibaev

**Affiliations:** ^1^ Key Laboratory for Plant Diversity and Biogeography of East Asia, Kunming Institute of Botany, Chinese Academy of Sciences, Kunming, Yunnan, China; ^2^ Yunnan International Joint Laboratory for Biodiversity of Central Asia, Kunming Institute of Botany, Chinese Academy of Sciences, Kunming, Yunnan, China; ^3^ Flora of Uzbekistan Laboratory, Institute of Botany of the Academy of Sciences of the Republic of Uzbekistan, Tashkent, Uzbekistan; ^4^ University of Chinese Academy of Sciences, Beijing, China; ^5^ International Joint Lab for Molecular Phylogeny and Biogeography, Institute of Botany, Academy Sciences of Uzbekistan, Tashkent, Uzbekistan

**Keywords:** *Hedysarum*, chloroplast genome, comparative analysis, phylogeny, protein-coding genes

## Abstract

*Hedysarum* is one of the largest genera in the Fabaceae family, mainly distributed in the Northern Hemisphere. Despite numerous molecular studies on the genus *Hedysarum*, there is still a lack of research aimed at defining the specific characteristics of the chloroplast genome (cp genome) of the genus. Furthermore, the interrelationships between sections in the genus based on the cp genome have not yet been studied. In this study, comprehensive analyses of the complete cp genomes of six *Hedysarum* species, corresponding to sections *Multicaulia*, *Hedysarum*, and *Stracheya* were conducted. The complete cp genomes of *H. drobovii*, *H. flavescens*, and *H. lehmannianum* were sequenced for this study. The cp genomes of six *Hedysarum* species showed high similarity with regard to genome size (except for *H. taipeicum*), gene sequences, and gene classes, as well as the lacking IR region. The whole cp genomes of the six species were found to contain 110 genes ranging from 121,176 bp to 126,738 bp in length, including 76 protein-coding genes, 4 rRNA genes, and 30 tRNA genes. In addition, chloroplast SSRs and repetitive sequence regions were reported for each species. The six *Hedysarum* species shared 7 common SSRs and exhibited 14 unique SSRs. As well, three highly variable genes (*clpP, accD*, and *atpF*) with high Pi values were detected among protein-coding genes. Furthermore, we conducted phylogenetic analyses using the complete cp genomes and 76 protein-coding genes of 14 legume species, including the seven *Hedysarum* species. The results showed that the *Hedysarum* species form a monophyletic clade closely related to the genera *Onobrychis* and *Alhagi*. Furthermore, both of our phylogenetic reconstructions showed that section *Stracheya* is more closely related to section *Hedysarum* than to section *Multicaulia*. This study is the first comprehensive work to investigate the genome characteristics of the genus *Hedysarum*, which provides useful genetic information for further research on the genus, including evolutionary studies, phylogenetic relationships, population genetics, and species identification.

## Introduction

*Hedysarum* L. is one of the large genera in the Fabaceae family, containing more than 160 species (Lock, 2005). Plants belonging to the genus *Hedysarum* are distributed in Eurasia, North Africa, and North America (Lock, 2005; Liu et al., 2017). The species occur in meadows, clayey and stony places, deserts, steppes, forests, tundra, river valleys, and mountain slopes (Choi and Ohashi, 2003). The genus *Hedysarum* includes perennial herbs and rarely semi-shrubs, which differ from closely related genera in pod and pollen morphology (Fedtschenko, 1948; Choi and Ohashi, 1996). Previous studies have shown that many species of the genus *Hedysarum* have been employed in traditional Chinese medicine to strengthen the immune system and improve the energy of the body (Dong et al., 2013).

Recent molecular studies have proposed to divide this genus into three main clades, largely corresponding to the sections *Hedysarum, Stracheya*, and *Multicaulia* (Amirahmadi et al., 2014; Nafisi et al., 2019). In these molecular phylogenetic studies, species were divided into three sections that were well supported, but intersection relationships remained unresolved, especially in section *Multicaulia*. This could be due to the selection of regions with low variability in the cp genome. Therefore, it is necessary to identify regions with high nucleotide diversity in the cp genome as molecular markers for future molecular phylogenetic studies of *Hedysarum*. Furthermore, previous studies have reported conflicting results regarding the phylogenetic relationship of section *Stracheya* with the other two sections within the *Hedysarum* genus. Duan et al. (2015) found that section *Stracheya* was closely related to section *Multicaulia* based on both nrDNA ITS and some plastid markers. However, Liu et al. (2017) reported that section *Stracheya* was placed together with section *Hedysarum* based on nrDNA ITS and plastid markers. Conversely, Nafisi et al. (2019) supported a closer relationship between section *Stracheya* and section *Multicaulia*. Therefore, determining the phylogenetic position of section *Stracheya* in the genus *Hedysarum* based on the cp genome is necessary.

Chloroplasts are important intracellular organelles, having an independent genome with several genes responsible for the process of photosynthesis in green algae and plants (Nazareno et al., 2015; Smith and Keeling, 2015; Yin et al., 2017). Most complete cp genomes harbor a typical quadripartite structure including a long single copy (LSC) region, a small single copy (SSC) region, and two copies of inverted repeat (IR) regions (Bock, 2007). *Hedysarum* belonging to the IRLC (Inverted Repeat Lacking Clade) clade is described by the lack of one copy of the inverted repeat (IR) region in the whole cp genome (Wojciechowski et al., 2004; She et al., 2019). Species belonging to the IRLC clade are characterized by having a cp genome size of around 121,000–133,000 bp (Moghaddam et al., 2022; Yuan et al., 2022; Tian et al., 2021). To date, the size of the cp genomes of only four *Hedysarum* species are known, including *H. petrovii*, *H. semenovii*, *H. polybotrys*, and *H. taipeicum*, with genome sizes of 122,571 bp, 123,407 bp, 122,232 bp, and 126,699 bp, respectively.

Comparative genomics can be used to identify important structural sequences and detect evolutionary changes across genomes since the comprehensive analysis of the cp genome of genera belonging to the IRLC clade such as *Astragalus*, *Onobrychis*, *Caragana*, and *Glycyrrhiza* have been reported (Kang et al., 2018; Moghaddam et al., 2022; Yuan et al., 2022; Tian et al., 2021). A detailed characterization of these species’ cp genome, including size, gene content, structure repeats, and GC content, as well as information about highly variable nucleotide regions, was provided. However, comprehensive studies on the genome structure of the genus *Hedysarum* have not been conducted so far.

In the present investigation, we detailed an overview of the complete sequence of the six *Hedysarum* species cp genome. We sequenced the complete cp genome of *H. drobovii*, *H. flavescens*, and *H. lehmannianum* to explore the relationships among *Hedysarum* species. We obtained the other three species (*H. petrovii*, *H. semenovii*, and *H. taipeicum*) from the National Center for Biotechnology Information (NCBI). The following questions were addressed: (1) what are the features of the cp genome of selected *Hedysarum* species? (2) How many potential microsatellite markers can the cp genome provide? (3) Which regions in the cp genome can be used as candidate molecular markers for future molecular phylogenetic studies? (4) What is the phylogenetic placement of section *Stracheya* within the genus *Hedysarum* based on the cp genome data?

## Materials and methods

### Plant materials

**Figure 1:**
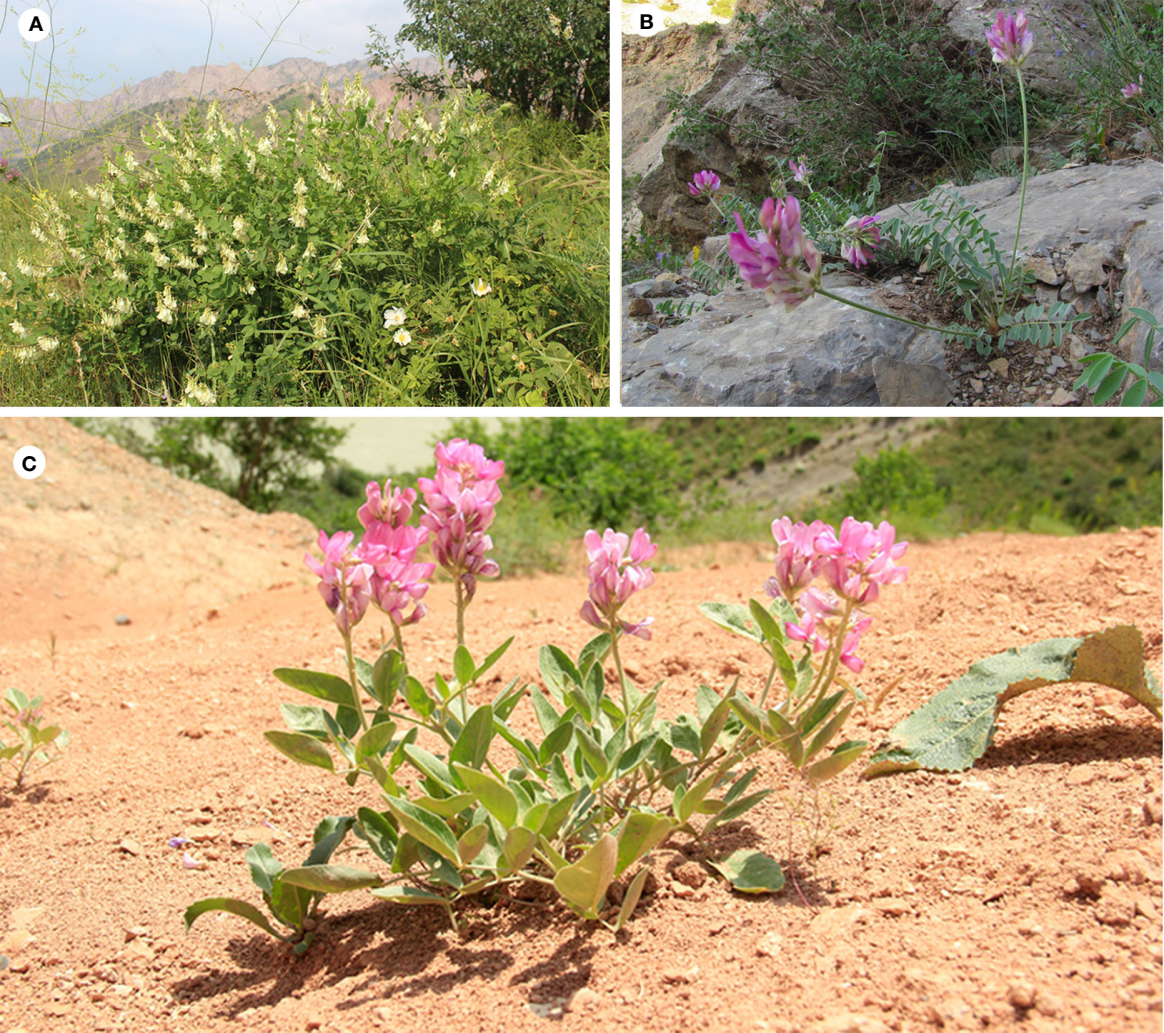
Three sequenced samples in this study. **(A)** *H. flavescens*, **(B)** *H. lehmaniannum*, and **(C)** *H. drobovii*. The photo of *H. lehmaniannum* was taken by O.Turginov.

For the comparative genome analysis, species from each section of *Hedysarum* were selected in this study: *Hedysarum drobovii* and *H. petrovii* from the *H*. sect. *Multicaulia*; *H. flavescens, H. semenovii*, and *H. taipeicum* from the *H*. sect. *Hedysarum*; *H. lehmaniannum* from the *H*. sect. *Stracheya*. Fresh material for *H. drobovii*, *H. flavescens*, and *H. lehmaniannum* was collected from Uzbekistan (*H. drobovii*: Western Tien Shan, Chatkal Range, E. 70.1045, N. 41.560301, altitude: 970 m a.s.l., 06 June 2020, *Dekhkonov, Ortiqov, Turdiev, Juramurodov 19062020117*; *H. flavescens*: Western Tien Shan, Chatkal Range, E. 70.019246 N. 41.508587, altitude: 2290 m a.s.l., 06 June 2020, *Dekhkonov, Ortiqov, Turdiev, Juramurodov 19062020089*; *H. lehmaniannum*: Hisar Range, Boysun district, E. 67.163574, N. 38.337148, altitude: 2390 m a.s.l., 13 June 2021, *Turginov, Rahmatov 13062021007*), and their complete chloroplast (cp) genome sequences were generated (**Figure 1**). Herbarium materials of these three species were stored in the National Herbarium of Uzbekistan (TASH).

### Sequencing, assembly, and annotation

Total genomic DNA was extracted from leaf material using DP305 Plant Genomic DNA kits (Tiangen, Beijing, China) following the manufacturer’s protocol. The sequencing library was generated using NEBNext^®^ Ultra^TM^ DNA Library Prep Kit for Illumina (NEB, USA, Catalog: E7370L) following the manufacturer’s recommendations, and index codes were added to each sample. Briefly, the genomic DNA sample was fragmented by sonication to a size of 350 bp. Then DNA fragments were end polished, A-tailed, and ligated with the full-length adapter for Illumina sequencing, followed by further PCR amplification. After, PCR products were purified by the AMPure XP system (Beverly, USA). Subsequently, the library quality was assessed on the Agilent 5400 system(Agilent, USA) and quantified by QPCR (1.5 nM). The qualified libraries were pooled and sequenced on Illumina platforms with PE150 strategy in Novogene Bioinformatics Technology Co., Ltd (Beijing, China), according to effective library concentrations and data amount required.

The resulting clean reads were assembled using the GetOrganelle pipeline (Jin et al., 2020) with the optimized parameters “-F plant_cp -w 0.6 -o -R 20 -t 8 -k 75,95,115,127 and”. Gene annotation was performed in Geneious v.10.0.2 and *H. polybotrys* (unpublished, accession number: MZ322397) was set as the reference. Start and stop codons and intron/exon boundaries for protein-coding genes were checked manually (Kearse et al., 2012).

### Simple sequence repeats

The chloroplast simple sequence repeats (SSRs) were identified using the MIcroSAtellite (MISA) web tool (Beier et al., 2017). The search conditions for SSRs were set to isolate perfect mono-, di-, tri-, tetra-, penta-, and hexa nucleotide motifs with a minimum of 10, 5, 4, 3, 3, and 3 repeats, respectively. The REPuter program (Kurtz et al., 2001) was used to identify repeats: forward, reverse, palindrome, and complement sequences in cp genomes. The following settings for repeat identification were used: (1) a hamming distance equal to three, (2) minimal repeat size set to 30 bp, and (3) maximum computed repeats set to 90 bp.

### Comparative analysis of chloroplast genomes

The cp genome was drawn using OGDRAWv1.1 (Lohse et al., 2007). Nucleotide variability (Pi) was calculated for the whole cp genome and protein-coding genes separately using DnaSP v. 6.12.03 software (Rozas et al., 2018). The window length was set to 800 bp and the step size was 200 bp. Furthermore, pairwise chloroplast genomic alignment among six species was compared by mVISTA in Shuffle-LAGAN mode (Frazer et al., 2004), and *H. polybotrys* (MZ322397) was used as a reference.

### Phylogenetic analysis

The three sequenced cp genomes of *Hedysarum* and 11 genomes from other species (including *Onobrychis gaubae, O. viciifolia, Caragana jubata, C. kozlowii, Oxytropis aciphylla*, and *O. glabra* as outgroups) retrieved from NCBI (**Supplementary Table 1**) were used to construct a phylogenetic tree. Phylogenetic tree reconstruction was performed using complete cp genomes and protein-coding sequences that were first aligned multiple times using MAFFT software (Katoh and Standley, 2013).

We reconstructed phylogenetic trees using Bayesian inference (BI), Maximum Parsimony (MP), and Maximum Likelihood (ML) methods. Nucleotide substitution models were selected statistically with the help of jModelTest2 on XSEDE (www.phylo.org) by considering the Akaike Information Criterion (AIC). The GTR+G model for the protein-coding sequences and the TVM+G model for the complete cp genomes were selected as the best model. For BI, we used MrBayes v. 3.2.7a (Ronquist et al., 2012) with 10 million generations with random trees sampled every 1000 generations. In the latter analysis, after discarding the first 25% of the trees as burn-in, a 50% majority-rule consensus tree was constructed from the remaining trees to estimate posterior probabilities (PP). The ML phylogeny was reconstructed using IQ-TREE 2.1.2 software (Minh et al., 2021) with 1000 bootstrap (BS) replicates to assess clade support (Nguyen et al., 2015). For MP analysis, we used PAUP* 4.0a169 (Swofford, 2002). The MP bootstrap analysis was performed with heuristic search, TBR branch-swapping, 1000 bootstrap replicates, random addition sequence with 10 replicates, and a maximum of 1000 trees saved per round.

## Results

### Chloroplast genome features of *Hedysarum* species

**Figure 2:**
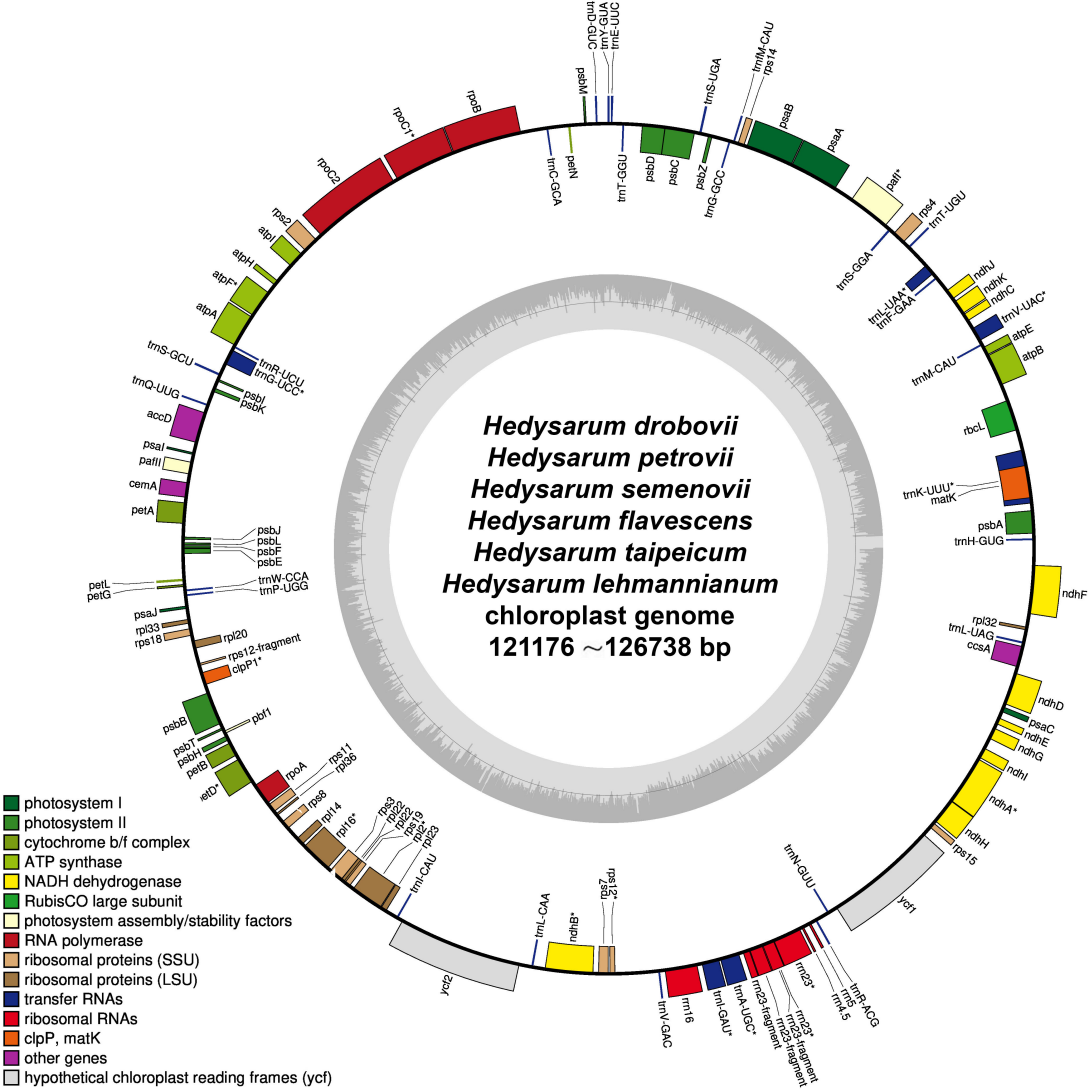
The chloroplast genome structure of six *Hedysarum* species. Genes shown outside the circles are transcribed clockwise, while those drawn inside are transcribed counterclockwise. Genes are color-coded according to their functional group.

The complete cp genomes of *H. drobovii*, *H. flavescens*, and *H. lehmannianum* were sequenced for this study. The sizes of the three newly sequenced species were 121,176 bp, 123,127 bp, and 123,586 bp, respectively. The *H. petrovii, H. semenovii*, and *H. taipeicum* species that were obtained from NCBI and the three newly sequenced species were without the typical quadripartite structure that contains a pair of IRs separated by LSC (large single-copy) and SSC (small single-copy) regions (**Figure 2**). The GC (guanine+cytosine) contents of the genomes of *H. drobovii*, *H. petrovii, H. flavescens*, *H. semenovii*, *H. lehmannianum*, and

*H. taipeicum* was 34.6%, 34.6%, 34.8%, 34.9%, 34.6%, and 35.1%, respectively. All six species’ genomes formed 110 genes including 76 protein-coding genes, 4 rRNA genes, and 30 tRNA genes (**Table 1**). A total of 16 genes in the cp genomes of six *Hedysarum* species consisted of introns, among which the genes *trnK-UUU, trnC-ACA, trnL-UAA, rpoC1, atpF, trnG-UCC, clpP, petB, petD, rpl16, rpl2, ndhB, trnE-UUC, trnA-UGC*, and *ndhA* each contained one intron, and only *ycf3* gene contained two introns (**Supplementary Table 2**). The *trnK-UUU* gene contained the largest intron, from 2407 (*H. petrovii*) to 2503 (*H. taipeicum*). Additionally, the *rps12* protein-coding gene is a trans-splicing gene that does not have introns in the 3’-end.

**Table 1:** List of genes annotated in the chloroplast genomes of six *Hedysarum* species in this study.

Category of genes	Group of genes	Genes
Genes for photosynthesis (44)	Subunits of photosystem I Subunits of photosystem II	*psaA, psaB, psaC, psaI, psaJ* *psbA, psbB, psbC, psbD, psbE, psbF, psbI, psbJ, psbH, psbK, psbL, psbM, psbN, psbT, psbZ*,
	Subunits of ATP synthase	*atpA, atpB, atpE, atpF, atpH, atpI*
	Subunits of NADH-dehydrogenase	*ndhA, ndhB, ndhC, ndhD, ndhE, ndhF, ndhG, ndhH, ndhI, ndhJ, ndhK*
	Subunits of cytochrome b/f complex	*petA, petB, petD, petG, petL, petN*
	RubisCO large subunit	*rbcL*
Self-replication (57)	Large subunit of ribosome	*rpl14, rpl16, rpl2, rpl20, rpl23, rpl32, rpl33, rpl36*
	Small subunit of ribosome	*rps2, rps3, rps4, rps7, rps8, rps11, rps12, rps14, rps15, rps18, rps19*
	RNA polymerase	*rpoA, rpoB, rpoC1, rpoC2*
	Ribosomal RNAs	*rrn5S, rrn4.5S, rrn16S, rrn23S*
	tRNA genes	*trnH-GUG, trnK-UUU, trnM-CAU, trnV-UAC, trnF-GAA, trnL-UAA, trnS-GGA, trnT-UGU, trnG-GCC, trnT-GGU, trnC-GCA, trnfM-CAU, trnS-UGA, trnE-UUC* (×2 or ×1)*, trnY-GUA, trnD-GUC, trnR-UCU, trnG-UCC, trnS-GCU, trnQ-UUG, trnW-CCA, trnP-UGG, trnI-CAU, trnL-CAA, trnN-GUU, trnL-UAG, trnV-GAC, trnA-UGC, trnI-GAU* (or *trnE-UUC), trnR-ACG*
Other genes (5)	Subunit of acetyl-CoA-carboxylase	*accD*
	c-type cytochrome synthesis gene	*ccsA*
	Envelop membrane protein	*cemA*
	Protease	*clpP*
	Maturase	*matK*
Genes with unknown functions (4)	hypothetical chloroplast reading frames (ycf)	*ycf1, ycf2, ycf3, ycf4*

### Repeat sequences and SSRs analysis

**Figure 3:**
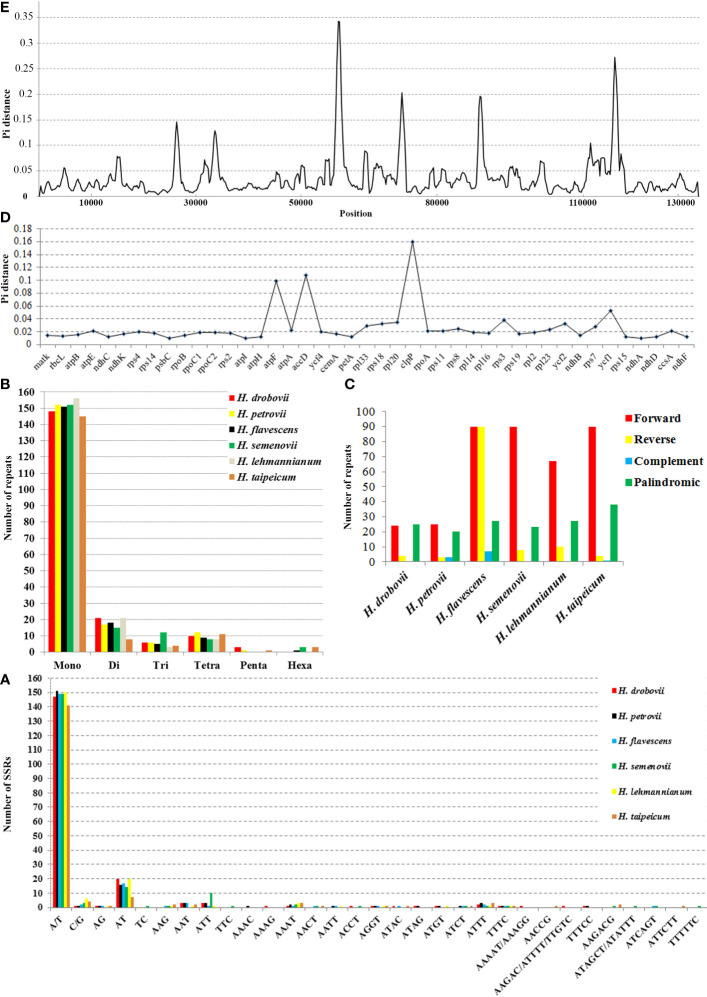
Chloroplast genome features of six *Hedysarum* species. Type of SSRs **(A)**; long repetitive sequences **(B)**; SSR distribution **(C)**. Nucleotide diversity (Pi) in protein-coding genes **(D)** and whole chloroplast genomes **(E)**. Among protein-coding genes, genes with nucleotide diversity < 0.01 are not shown.

A total of 188 SSRs were detected using the MISA web tool in the cp genome of each *H. drobovii*, *H. petrovii*, and *H. lehmannianum* species, while *H. flavescens*, *H. semenovii*, and *H. taipeicum* had different SSRs of 184, 190, and 172, respectively. Among six *Hedysarum* cp genomes, the most abundant repeats were the mononucleotides from 145 (*H. taipeicum*) to 156 (*H. lehmannianum*), and the most dominant SSR was A/T (**Figures 3A, B**). Di-nucleotides (especially AT) were the second most predominant, varying from eight (*H. taipeicum*) to 21 (*H. lehmannianum*). A high number of trinucleotides was detected in *H. semenovii* (12), whereas a low number of trinucleotides was in *H. lehmannianum* (3). A total of 57 repeats of tetranucleotides, varying from seven (*H. drobovii*) to 12 (*H. petrovii*) were identified among the six *Hedysarum* cp genomes. Our analysis identified five pentanucleotide repeats in three *Hedysarum* species: *H. drobovii* (AAAAT, AAAGG, and AAGAC), *H. petrovii* (TTTCC), and *H. taipeicum* (AACCG), while the remaining three species did not exhibit any pentanucleotide repeats. Additionally, hexanucleotide repeats were detected only in *H. flavescens* (ATCAGT), *H. semenovii* (AAGACG, ATAGCT, and ATATTT), and *H. taipeicum* (AAGACG(× 2) and ATTCTT).

In our study, we examined common and unique SSRs in six *Hedysarum* species (**Supplementary Tables 3, 4**), and we found that the majority of repeat units were composed of A and T, with rare occurrences of C or G, indicating that the SSRs of different species had an obvious bias in the base types of repeat units. Common SSRs included A, G, T, AT, AAAT, ATTT, and TTTC, which were present in all six species. We also identified 14 unique SSRs, including AAAG, AAAGG, AAAAT, AAGAC, ATTTT, and TTGTC in *H. drobovii*; TC, TTC, ATAGCT, and TTTTTC in *H. semenovii*; and AACCG and ATTCTT in *H. taipeicum*. Only one AAAC SSR was detected in *H. petrovii*, while no unique SSRs were identified in *H. flavescens* and *H. lehmannianum*.

In this study, we found many repeat regions including forward, reverse, palindromic, and complementary repeats (**Figure 3C**). Among the six studied *Hedysarum* species, the longest repetitive sequences were detected in the *H. flavescens* cp genome, which had 214 repetitive sequences with lengths of no more than 48 bp. On the contrary, the smallest repetitive sequences were found in *H. drobovii* and *H. petrovii* cp genomes, of which 53 and 51 scattered repetitive sequences with lengths of no more than 18 bp and 13 bp, respectively. The length of the largest forward and palindromic repeats were 62 bp and 36 bp in the *H. taipeicum* cp genome, respectively, whereas the largest reverse and complement repeat lengths were 48 bp and 7 bp in the *H. flavescens* cp genome, respectively. Equal numbers of forward repeats (90) were detected in *H. flavescens*, *H. semenovii*, and *H. taipeicum.* Additionally, the complement repeat was not found in the cp genomes of *H. drobovii, H. semenovii*, and *H. lehmannianum*.

### Comparative genomic divergence and hotspot regions

We calculated the nucleotide diversity (Pi) values to estimate the divergence levels of the whole cp genome and protein-coding genes of the six *Hedysarum* species (**Figures 3D, E**). The most high-variation regions (Pi=0.3425) of the whole *Hedysarum* cp genomes were mainly concentrated between 55000 bp and 60000 bp. According to the Pi value results of protein-coding genes of six *Hedysarum* species, *clpP* (0.16), *accD* (0.108), and *atpF* (0.099) genes had the highest variability, while the *psaC* gene had a low nucleotide diversity (0.00136). In addition, the Pi values were less than 0.01 in 43.4% of the total protein-coding genes, while in 39.5% were 0.01–0.02. Only 26.3% of total protein-coding genes had Pi > 0.02 (**Supplementary Table 5**).

**Figure 4:**
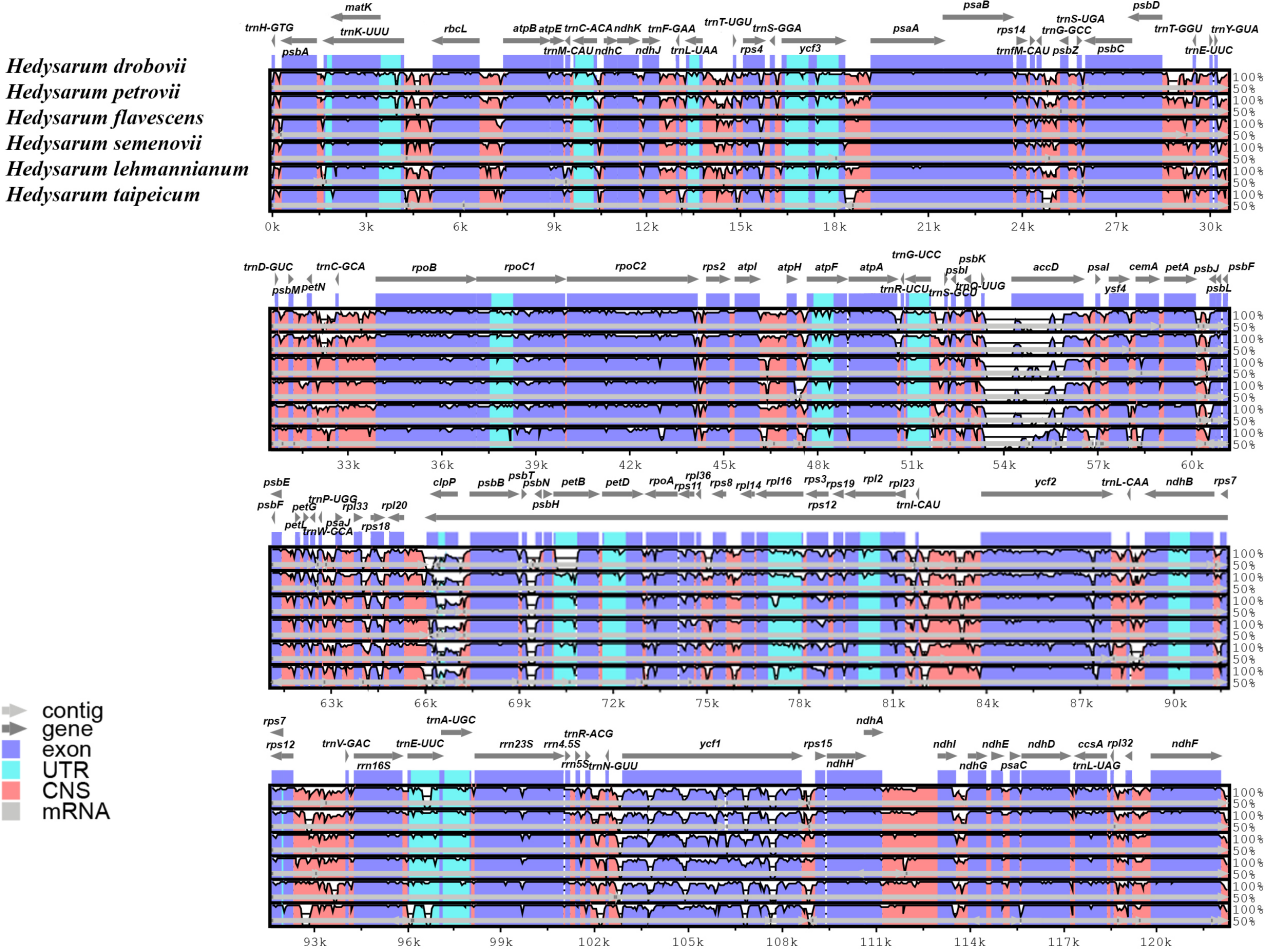
Alignment visualization of the chloroplast genome sequences of six *Hedysarum* species using mVISTA. Annotated genes are shown along the top. Genomic regions are color-coded to indicate protein-coding regions, exons, UTRs, and CNS. The similarity among the chloroplast genomes is shown on a vertical scale ranging from 50 to 100%.

The cp genome sequences of six *Hedysarum* species were compared using the mVISTA software, and their alignments were visualized with annotation data (**Figure 4**). According to this visualization analysis, differences among sequences occurred in *clpP*, *accD*, and *ycf1* genes from the coding regions and mainly in non-coding intergenic regions. Encoded gene classes and alignments of the main part of the coding regions among the six *Hedysarum* were highly congruous.

### Phylogenetic analysis

**Figure 5:**
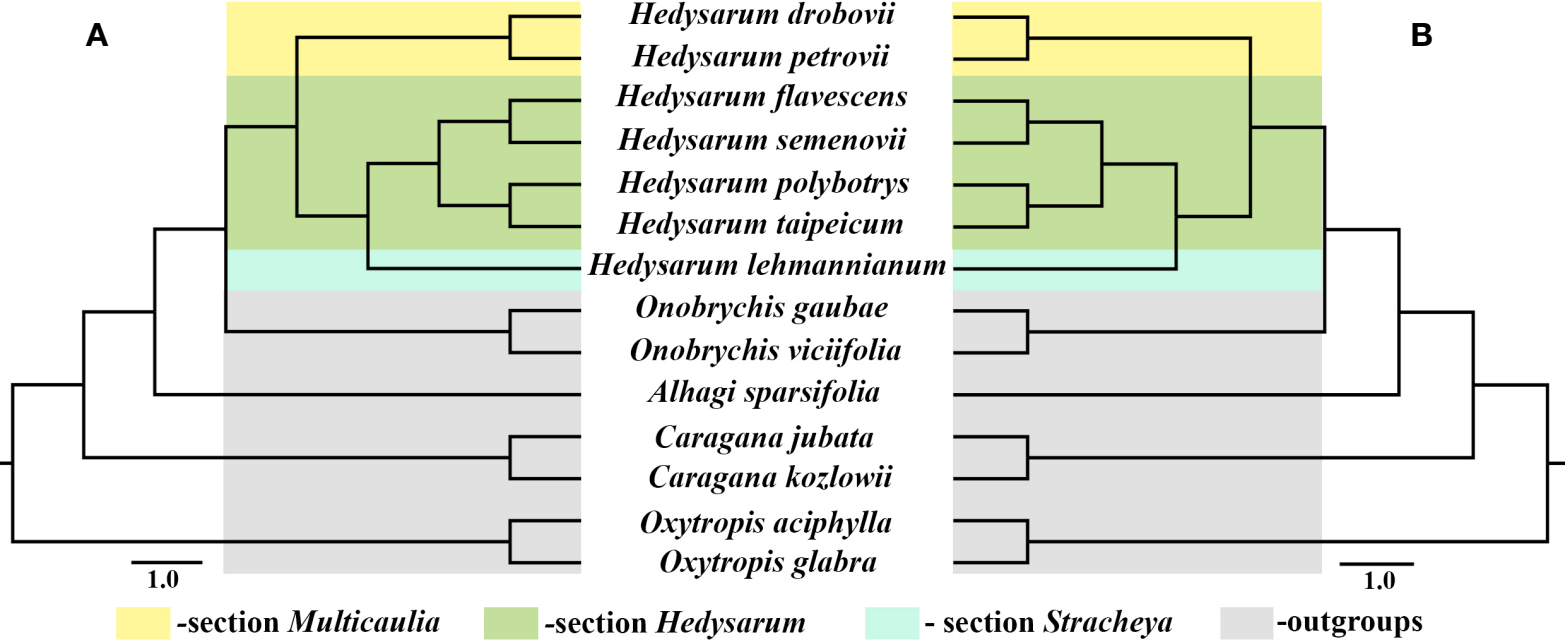
Phylogenetic tree of 14 species including seven *Hedysarum* species using BI, ML, and MP analyses based on complete cp genomes **(A)** and their 76 protein-coding genes **(B)**. All branches were maximally supported by BI (1.00), ML (100%), and MP (100%) methods.

Seven *Hedysarum* and seven related cp genome data were analyzed phylogenetically. Phylogenetic reconstructions based on the complete cp genome and protein-coding genes yielded similar results (**Figures 5A, B**). All clades in both trees were strongly supported by BI, ML, and MP analyses, with 1.00, 100%, and 100% bootstrap values, respectively. Additionally, the results of the phylogenetic analysis based on the complete cp genomes and 76 protein-coding genes showed that *Hedysarum* was monophyletic. The clade including species of the genus *Onobrychis* was sister to *Hedysarum*. The *Hedysarum* species used in this study were formed into two clades. One is a clade containing *H. drobovii* and *H. petrovii* species corresponding to section *Multicaulia*. The second clade was formed by five *Hedysarum* species. *H. flavesens, H. semenovii, H. taipeicum*, and *H. polybotrys* species were placed into the section *Hedysarum*, and *H. lehmannianum* belonged to the section *Stracheya*, which was sister to the section *Hedysarum*.

## Discussion

This study is the first to comprehensively examine the features of cp genomes in *Hedysarum* species. We compared the cp genomes of six species belonging to three sections that were distributed in different regions. We sequenced the cp genome of *H. drobovii, H. flavescens*, and *H. lehmannianum* for this study. The sizes of the six cp genomes ranged from 121,176 bp (*H. drobovii*) to 126,738 bp (*H. taipeicum*). It is worth noting that many related genera with similar cp genome sizes to *Hedysarum* have been reported in recent years (Tian et al., 2021; Bei et al., 2022; Moghaddam et al., 2022).

The cp genomes of *Hedysarum* species have 110 genes, including 76 protein-coding genes, 30 transfer RNA genes, and 4 ribosomal RNA genes. The structural composition of *Hedysarum* cp genomes revealed similarity with other IRLC clade species (Su et al., 2019; Bei et al., 2022; Moghaddam et al., 2022). The cp genomes of six *Hedysarum* species showed high similarity with regard to genome size (except for *H. taipeicum* which was 126,738 bp), gene sequences, gene classes, and the lacking IR region. All selected *Hedysarum* species were found to have lost one copy of the IR region, which was first identified in *H. taipeicum* by She et al. (2019). This loss of the IR region is common in most species belonging to the subfamily Papilionoideae in the family Fabaceae, forming a clade named the IR-lacking clade (IRLC) (Wojciechowski et al., 2004). The GC content of the six *Hedysarum* species in this study was highly similar, which is an important indicator of species affinity according to Tamura et al. (2011).

Introns are recognized as being central to the regulation of gene expression in plants and animals (Callis et al., 1987; Emami et al., 2013: Choi et al., 1991). In the present study, 15 genes with one intron and one gene (*ycf3*) with two introns were identified in each of the cp genomes of the six studied *Hedysarum* species. Most of the 16 identified genes have a high similarity in the structure of introns. However, a structural change was detected in the intron of the *petB* and *clpP* genes of *H. drobovii* and *H. lehmannianum*, respectively. The intron of the *petB* gene in the cp genome of *H. drobovii* is very short (9 bp); whereas, in the other five species, it ranges from 806 bp (*H. flavescens*) to 864 bp (*H. taipeicum*). Similarly, the intron of the *clpP* gene in the cp genome of *H. lehmannianum* is 6 bp long, while in other species it is from 159 bp (*H flavescens*) to 613 bp (*H. taipeicum*). However, the implications or link between gene expression and short or long introns for *Hedysarum* have not been studied. Further experimental work on the roles of introns in *Hedysarum* is therefore essential and should prove interesting. In consonance with previous studies, the *trnK-UUU* gene in the *Hedysarum* cp genome was observed to be harboring the largest intron (2407-2503 bp) which includes the *matK* gene.

The chloroplast SSRs were used in evolutionary studies, phylogenetic relationships, and plant population genetics and species identification as molecular markers (Olmstead and Palmer, 1994; Saski et al., 2005). A total of 172 SSRs (*H. taipeicum*) to 190 SSRs (*H. semenovii*) were found in the cp genome of six *Hedysarum* species. Several studies found that the mononucleotide repeats were dominant among SSRs in the cp genome, where A/T bases account for the majority (Ellegren, 2004; George et al., 2015; Ren et al., 2021). Likewise, A/T mononucleotide repeats were dominant among SSRs in the six *Hedysarum* cp genomes, ranging from 78.7% to 84.3%. Furthermore, the identified common and unique SSRs might play an important role in the analysis of the genetic diversity of the genus *Hedysarum*. In particular, the unique pentanucleotide SSRs identified in *H. drobovii* (AAAGG, AAAAT, AAGAC, ATTTT, and TTGTC) and *H. taipeicum* (AACCG), as well as the unique hexanucleotide SSRs identified in *H. semenovii* (ATAGCT and TTTTTC) and *H. taipeicum* (ATTCTT) may be effectively utilized in the future for species identification and assessment of genetic diversity in their populations. In addition, repeat sequences are known to play an important role in cp genome rearrangement, recombination, gene duplication, deletion, and gene expression (Gemayel et al., 2010; Do et al., 2014; Vieira et al., 2014; Li and Zheng, 2018),. They also have been reported to be responsible for substitutions and indels in the cp genome (Yi et al., 2013). We identified 51 (*H. petrovii*) to 214 (*H. flavescens*) repeat sequences among the six *Hedysarum* cp genomes analyzed, with forward repeats being the most common in *H. petrovii*, *H. flavescens*, *H. semenovii*, *H. lehmannianum*, and *H. taipeicum*, whereas palindromic and forward repeats were the most abundant in *H. drobovii*. Notably, *H. drobovii* and *H. petrovii*, both belonging to section *Multicaulia*, had significantly fewer repeat regions (53 and 51, respectively) compared to the other species (104–214). Further investigation into repeat sequences in section *Multicaulia* is necessary.

DNA barcodes with high variability are crucial for species identification, resource conservation, and phylogenetic analyses (Gregory, 2005; Bringloe and Saunders, 2019; Chen et al., 2019; Liu et al., 2019). Our comparison of *Hedysarum* species’ cp genomes revealed high similarity in gene content and gene order, with genome lengths ranging from 121,176 to 126,738 bp. However, mVISTA analyses indicated that sequence variation was higher in non-coding regions than in other regions. Nucleotide diversity analysis identified six highly variable regions in the whole cp genome of *Hedysarum* species, mainly located in non-coding regions. Three protein-coding genes, *clpP*, *accD*, and *atpF*, exhibited higher Pi values and were found to be highly variable regions. The variability of the *clpP* gene can be attributed to the large variation in its Exon I length between species, ranging from 3 bp (*H. drobovii*) to 219 bp (*H. taipeicum* and *H. lehmannianum*) (**Supplementary Table 2**). The *clpP* and *accD* genes have been reported to play an important role in counteracting biotic and abiotic stress (Singh et al., 2015; Sinha et al., 2018; Ali and Baek, 2020), while the *atpF* gene is involved in the synthesis of ATF during photosynthesis (Ghulam et al., 2012), which is greatly influenced by altitude conditions (Wang et al., 2017). The high Pi values observed in these genes may reflect adaptation to different environmental conditions. Moreover, these highly variable regions can serve as candidate molecular markers and a reference for identifying future *Hedysarum* species. The *clpP, accD*, and *atpF* gene exon regions have similarly been identified as some of the most highly variable hotspot regions in cp genomes of some species (Mo et al., 2020; Mascarello et al., 2021; Moghaddam et al., 2022; Long et al., 2023).

Our phylogenetic analysis based on complete cp genomes and protein-coding genes confirmed previous studies on cp genome data of IRLC clade species, determining the phylogenetic position of *Hedysarum* as a sister to *Onobrychis* (She et al., 2019; Jin et al., 2021; Moghaddam et al., 2022; Tian et al., 2021). Our study also confirms the monophyly of *Hedysarum* based on plastid DNA genes, which is consistent with previous studies by Duan et al. (2015); Liu et al. (2017), and Nafisi et al. (2019). Although a limited number of species were used in our study, the phylogenetic relationships among the three sections of *Hedysarum* were analyzed using complete cp genomes and 76 protein-coding genes for the first time. Our results suggested that sections of *Hedysarum* could be monophyletic based on both cp genome data. However, further studies with more species, particularly from section *Stracheya*, are necessary to confirm this outcome. Furthermore, both our phylogenetic reconstructions revealed a close relationship between section *Stracheya* and *Hedysarum*, which is consistent with previous findings by Liu et al. (2017), but incongruous with the outcomes of Duan et al. (2015) and Nafisi et al. (2019). Additionally, this relationship is supported by the shared morphological characteristics between species of section *Stracheya* and *Hedysarum*, including leaves with numerous leaflets (4-15 paired), wings longer than half of the keel, and pods lacking ribs, bristles, or spines.

## Conclusion

Our study is the first research work to investigate the genome characteristics of the genus *Hedysarum*. We sequenced, assembled, and annotated the cp genome of *H. drobovii*, *H. flavescens* va *H. lehmannianum* using high-throughput technology. Our study is based on cp genome data from a total of six *Hedysarum* species, including three previously published species. The cp genomes of all six *Hedysarum* species analyzed contained 110 genes, including 76 protein-coding genes, 4 rRNA genes, and 30 tRNA genes. We identified between 172 and 190 microsatellites and 51 to 214 pairs of repeat sequences among the six *Hedysarum* species cp genomes. In addition, we identified seven common SSRs and 14 unique SSRs in the studied *Hedysarum* species. Furthermore, we detected highly variable regions in the *clpP*, *accD*, and *atpF* protein-coding genes. These repeat motifs and highly variable genes could be used for evolutionary studies, phylogenetic relationships, plant population genetics, and species identification. Our phylogenetic reconstructions using the complete cp genome and protein-coding genes confirmed the monophyly of *Hedysarum*. Additionally, we supported the close relationship between section *Stracheya* and section *Hedysarum* using all three BI, ML, and MP methods. However, future studies using more species will provide a better understanding of the relationships among *Hedysarum* sections.

## Data availability statement

The datasets presented in this study can be found in online repositories. The names of the repository/repositories and accession number(s) can be found in the article/**Supplementary Material**.

## Author contributions

IJ: Conceptualization, methodology, data analysis, identification, visualization, writing, original draft preparation, reviewing, editing, and discussing. DM: methodology and data analysis. ZY: methodology and collection. KT: Supervision, investigation, identification, reviewing, editing, and discussing. All authors contributed to the article and approved the submitted version.
