# Supplementary material for: First comparative analysis of complete chloroplast genomes among six Hedysarum (Fabaceae) species
Source: Front Plant Sci. 2023 Aug 18;14:1211247. doi: 10.3389/fpls.2023.1211247 (PMC10473476; doi:10.3389/fpls.2023.1211247)
Supplement: Supplementary file 1 [file Table_1.doc]

**Table S1 Species used for polygenetic analysis. New sequences are marked with an asterisk**

| **No** | **Species** | **Genome size (bp)** | **GenBank accession numbers** |
| --- | --- | --- | --- |
| **1** | *Hedysarum drobovii* Korotkova* | 121,176 | OQ736739 |
| **2** | *Hedysarum flavescens* Regel & Schmalh.* | 123,127 | OQ736732 |
| **3** | *Hedysarum lehmannianum* Bunge* | 123,586 | OQ736747 |
| **4** | *Hedysarum petrovii* Yakovlev | 122,571 | MT120797 |
| **5** | *Hedysarum semenovii* Regel & Herder | 123,407 | ON009074 |
| **6** | *Hedysarum taipeicum* (Hand.-Mazz.) K.T.Fu | 126,699 | MK426698 |
| **7** | *Hedysarum polybotrys* Hand.-Mazz. | 122,232 | MZ322397 |
| **8** | *Onobrychis gaubae*  Bornm*.* | 122,688 | LC647182 |
| **9** | *Onobrychis viciifolia*  Scop*.* | 121,932 | NC_053934 |
| **10** | *Alhagi sparsifolia* Shap*.* | 128,233 | MW349013 |
| **11** | *Caragana jubata* (Pall.) Poir. | 128,132 | MT211963 |
| **12** | *Caragana kozlowii* Kom*.* | 131,274 | MG386382 |
| **13** | *Oxytropis aciphylla*  Ledeb*.* | 122,121 | OK143433 |
| **14** | *Oxytropis glabra* DC. | 122,094 | MW349014 |
