# Supplementary material for: First comparative analysis of complete chloroplast genomes among six Hedysarum (Fabaceae) species
Source: Front Plant Sci. 2023 Aug 18;14:1211247. doi: 10.3389/fpls.2023.1211247 (PMC10473476; doi:10.3389/fpls.2023.1211247)
Supplement: Supplementary file 2 [file Table_2.doc]

**Table S2 Location and length of intron-containing genes in six *Hedysarum* species**

| ***Hedysarum drobovii*** | | | | | | | |
| --- | --- | --- | --- | --- | --- | --- | --- |
| **Gene** | **Start** | **End** | **ExonI** | **IntronI** | **ExonII** | **IntronII** | **ExonIII** |
| ***trnK-UUU*** | 1696 | 4177 | 29 | 2417 | 65 |  |  |
| ***trnC-ACA*** | 9546 | 10236 | 40 | 616 | 35 |  |  |
| ***trnL-UAA*** | 13279 | 13784 | 51 | 420 | 35 |  |  |
| ***ycf3*** | 16374 | 18336 | 126 | 719 | 228 | 737 | 153 |
| ***rpoC1*** | 37401 | 40216 | 432 | 760 | 1625 |  |  |
| ***atpF*** | 48097 | 49368 | 168 | 693 | 411 |  |  |
| ***trnG-UCC*** | 51202 | 51927 | 48 | 655 | 23 |  |  |
| ***clpP*** | 66299 | 66985 | 3 | 323 | 363 |  |  |
| ***petB*** | 69861 | 70508 | 5 | 9 | 634 |  |  |
| ***petD*** | 70704 | 71909 | 8 | 723 | 475 |  |  |
| ***rpl16*** | 75701 | 77178 | 360 | 1109 | 9 |  |  |
| ***rpl2*** | 78661 | 80180 | 435 | 716 | 369 |  |  |
| ***ndhB*** | 88251 | 90418 | 762 | 683 | 723 |  |  |
| ***trnE-UUC*** | 95467 | 96297 | 33 | 769 | 29 |  |  |
| ***trnA-UGC*** | 96334 | 97262 | 38 | 884 | 47 |  |  |
| ***ndhA*** | 109362 | 111645 | 552 | 1192 | 540 |  |  |

| ***Hedysarum petrovii*** | | | | | | | |
| --- | --- | --- | --- | --- | --- | --- | --- |
| **Gene** | **Start** | **End** | **ExonI** | **IntronI** | **ExonII** | **IntronII** | **ExonIII** |
| ***trnK-UUU*** | 1703 | 4174 | 29 | 2407 | 36 |  |  |
| ***trnC-ACA*** | 9495 | 10185 | 40 | 616 | 35 |  |  |
| ***trnL-UAA*** | 13220 | 13717 | 51 | 412 | 35 |  |  |
| ***ycf3*** | 16301 | 18273 | 126 | 718 | 228 | 748 | 153 |
| ***rpoC1*** | 37820 | 40625 | 432 | 751 | 1623 |  |  |
| ***atpF*** | 48495 | 49766 | 168 | 693 | 411 |  |  |
| ***trnG-UCC*** | 51622 | 52347 | 48 | 655 | 23 |  |  |
| ***clpP*** | 66930 | 67501 | 12 | 197 | 363 |  |  |
| ***petB*** | 70383 | 71847 | 6 | 817 | 642 |  |  |
| ***petD*** | 72045 | 73250 | 8 | 723 | 475 |  |  |
| ***rpl16*** | 77111 | 78589 | 360 | 1110 | 9 |  |  |
| ***rpl2*** | 80009 | 81548 | 435 | 712 | 393 |  |  |
| ***trnE-UUC*** | 96595 | 97645 | 33 | 989 | 29 |  |  |
| ***trnA-UGC*** | 97682 | 98609 | 38 | 843 | 47 |  |  |
| ***ndhB*** | 89588 | 91755 | 762 | 683 | 723 |  |  |
| ***ndhA*** | 110775 | 113063 | 552 | 1197 | 540 |  |  |

| ***Hedysarum flavescens*** | | | | | | | |
| --- | --- | --- | --- | --- | --- | --- | --- |
| **Gene** | **Start** | **End** | **ExonI** | **IntronI** | **ExonII** | **IntronII** | **ExonIII** |
| ***trnK-UUU*** | 1696 | 4221 | 29 | 2460 | 37 |  |  |
| ***trnC-ACA*** | 9703 | 10398 | 40 | 616 | 40 |  |  |
| ***trnL-UAA*** | 13408 | 13914 | 51 | 421 | 35 |  |  |
| ***ycf3*** | 16607 | 18612 | 126 | 728 | 228 | 771 | 153 |
| ***rpoC1*** | 37684 | 40507 | 432 | 757 | 1635 |  |  |
| ***atpF*** | 48361 | 49623 | 168 | 681 | 414 |  |  |
| ***trnG-UCC*** | 51568 | 52312 | 48 | 674 | 23 |  |  |
| ***clpP*** | 67269 | 67856 | 66 | 159 | 363 |  |  |
| ***petB*** | 70766 | 72219 | 6 | 806 | 642 |  |  |
| ***petD*** | 72414 | 73608 | 8 | 712 | 475 |  |  |
| ***rpl16*** | 77345 | 78794 | 360 | 1081 | 9 |  |  |
| ***rpl2*** | 80223 | 81741 | 435 | 694 | 390 |  |  |
| ***trnE-UUC*** | 96932 | 97982 | 33 | 989 | 29 |  |  |
| ***trnA-UGC*** | 98019 | 98942 | 38 | 839 | 47 |  |  |
| ***ndhB*** | 89755 | 90516 | 762 | 678 | 723 |  |  |
| ***ndhA*** | 111098 | 1205 | 552 | 1205 | 540 |  |  |

| ***Hedysarum semenovii*** | | | | | | | |
| --- | --- | --- | --- | --- | --- | --- | --- |
| **Gene** | **Start** | **End** | **ExonI** | **IntronI** | **ExonII** | **IntronII** | **ExonIII** |
| ***trnK-UUU*** | 1686 | 4206 | 29 | 2521 | 37 |  |  |
| ***trnC-ACA*** | 9735 | 10430 | 40 | 616 | 40 |  |  |
| ***trnL-UAA*** | 13466 | 13972 | 51 | 421 | 35 |  |  |
| ***ycf3*** | 16666 | 18659 | 126 | 727 | 228 | 766 | 147 |
| ***rpoC1*** | 37952 | 40770 | 432 | 752 | 1635 |  |  |
| ***atpF*** | 48516 | 49786 | 168 | 692 | 411 |  |  |
| ***trnG-UCC*** | 51732 | 52474 | 48 | 672 | 23 |  |  |
| ***clpP*** | 67462 | 68035 | 15 | 196 | 363 |  |  |
| ***petB*** | 70951 | 72406 | 6 | 808 | 642 |  |  |
| ***petD*** | 72600 | 73794 | 8 | 712 | 475 |  |  |
| ***rpl16*** | 77540 | 79007 | 360 | 1099 | 9 |  |  |
| ***rpl2*** | 80431 | 81940 | 435 | 695 | 390 |  |  |
| ***trnE-UUC*** | 97615 | 98670 | 33 | 994 | 29 |  |  |
| ***trnA-UGC*** | 98707 | 99630 | 38 | 839 | 47 |  |  |
| ***ndhB*** | 90376 | 92538 | 762 | 678 | 723 |  |  |
| ***ndhA*** | 111776 | 114078 | 552 | 1211 | 540 |  |  |

| ***Hedysarum lehmannianum*** | | | | | | | |
| --- | --- | --- | --- | --- | --- | --- | --- |
| **Gene** | **Start** | **End** | **ExonI** | **IntronI** | **ExonII** | **IntronII** | **ExonIII** |
| ***trnK-UUU*** | 1766 | 4261 | 29 | 2431 | 36 |  |  |
| ***trnC-ACA*** | 9834 | 10528 | 40 | 615 | 40 |  |  |
| ***trnL-UAA*** | 13638 | 14144 | 51 | 421 | 35 |  |  |
| ***ycf3*** | 16816 | 18791 | 126 | 725 | 228 | 744 | 153 |
| ***rpoC1*** | 38086 | 40894 | 432 | 754 | 1623 |  |  |
| ***atpF*** | 48716 | 49980 | 168 | 686 | 411 |  |  |
| ***trnG-UCC*** | 51930 | 52669 | 48 | 669 | 23 |  |  |
| ***clpP*** | 67386 | 67973 | 219 | 6 | 363 |  |  |
| ***petB*** | 70903 | 72355 | 6 | 826 | 621 |  |  |
| ***petD*** | 72548 | 73742 | 8 | 712 | 475 |  |  |
| ***rpl16*** | 77459 | 78931 | 360 | 1104 | 9 |  |  |
| ***rpl2*** | 80386 | 81906 | 435 | 693 | 393 |  |  |
| ***trnE-UUC*** | 96895 | 97944 | 33 | 988 | 29 |  |  |
| ***trnA-UGC*** | 97981 | 98894 | 38 | 829 | 47 |  |  |
| ***ndhB*** | 90039 | 92201 | 762 | 678 | 723 |  |  |
| ***ndhA*** | 111269 | 113559 | 552 | 1199 | 540 |  |  |

| ***Hedysarum taipeicum*** | | | | | | | |
| --- | --- | --- | --- | --- | --- | --- | --- |
| **Gene** | **Start** | **End** | **ExonI** | **IntronI** | **ExonII** | **IntronII** | **ExonIII** |
| ***trnK-UUU*** | 2131 | 4699 | 29 | 2503 | 37 |  |  |
| ***trnC-ACA*** | 10181 | 10876 | 40 | 616 | 40 |  |  |
| ***trnL-UAA*** | 13934 | 14570 | 51 | 551 | 35 |  |  |
| ***ycf3*** | 17128 | 19130 | 126 | 720 | 228 | 776 | 153 |
| ***rpoC1*** | 38364 | 41170 | 432 | 710 | 1665 |  |  |
| ***atpF*** | 49181 | 50465 | 168 | 692 | 426 |  |  |
| ***trnG-UCC*** | 52569 | 53401 | 48 | 762 | 23 |  |  |
| ***clpP*** | 69212 | 70400 | 219 | 613 | 363 |  |  |
| ***petB*** | 73392 | 74903 | 6 | 864 | 642 |  |  |
| ***petD*** | 75097 | 76291 | 8 | 712 | 475 |  |  |
| ***rpl16*** | 80196 | 81607 | 360 | 1043 | 9 |  |  |
| ***rpl2*** | 83102 | 84616 | 435 | 690 | 390 |  |  |
| ***trnE-UUC*** | 99854 | 100744 | 33 | 829 | 29 |  |  |
| ***trnA-UGC*** | 100781 | 101704 | 38 | 839 | 47 |  |  |
| ***ndhB*** | 92858 | 95015 | 762 | 673 | 723 |  |  |
| ***ndhA*** | 114556 | 116910 | 552 | 1263 | 540 |  |  |
