# Supplementary material for: First comparative analysis of complete chloroplast genomes among six Hedysarum (Fabaceae) species
Source: Front Plant Sci. 2023 Aug 18;14:1211247. doi: 10.3389/fpls.2023.1211247 (PMC10473476; doi:10.3389/fpls.2023.1211247)
Supplement: Supplementary file 3 [file Table_3.doc]

**Supplementary Table 3. Common Simple sequence repeats (SSRs) in the chloroplast genome (cpDNA) of six *Hedysarum* species**

| SSRs | *H. drobovii* | *H. flavescens* | *H. lehmannianum* | *H. petrovii* | *H. semenovii* | *H. taipeicum* |
| --- | --- | --- | --- | --- | --- | --- |
| A | 78 | 82 | 78 | 79 | 83 | 82 |
| AAAT | 1 | 1 | 3 | 2 | 2 | 3 |
| AT | 20 | 17 | 20 | 16 | 14 | 7 |
| ATTT | 2 | 2 | 1 | 3 | 1 | 3 |
| G | 1 | 1 | 3 | 1 | 2 | 3 |
| T | 69 | 67 | 72 | 72 | 66 | 59 |
| TTTC | 1 | 1 | 1 | 1 | 1 | 1 |
