# Supplementary material for: First comparative analysis of complete chloroplast genomes among six Hedysarum (Fabaceae) species
Source: Front Plant Sci. 2023 Aug 18;14:1211247. doi: 10.3389/fpls.2023.1211247 (PMC10473476; doi:10.3389/fpls.2023.1211247)
Supplement: Supplementary file 4 [file Table_4.doc]

**Supplementary Table 4. Unique Simple sequence repeats (SSRs) in the chloroplast genome (cpDNA) of six *Hedysarum* species**

| SSRs | *H. drobovii* | *H. flavescens* | *H. lehmannianum* | *H. petrovii* | *H. semenovii* | *H. taipeicum* |
| --- | --- | --- | --- | --- | --- | --- |
| AAAAT | 1 | 0 | 0 | 0 | 0 | 0 |
| AAAC | 0 | 0 | 0 | 1 | 0 | 0 |
| AAAG | 1 | 0 | 0 | 0 | 0 | 0 |
| AAAGG | 1 | 0 | 0 | 0 | 0 | 0 |
| AACCG | 0 | 0 | 0 | 0 | 0 | 1 |
| AAGAC | 1 | 0 | 0 | 0 | 0 | 0 |
| ATAGCT | 0 | 0 | 0 | 0 | 1 | 0 |
| ATATTT | 0 | 0 | 0 | 0 | 1 | 0 |
| ATTCTT | 0 | 0 | 0 | 0 | 0 | 1 |
| ATTTT | 1 | 0 | 0 | 0 | 0 | 0 |
| TC | 0 | 0 | 0 | 0 | 1 | 0 |
| TTC | 0 | 0 | 0 | 0 | 1 | 0 |
| TTGTC | 1 | 0 | 0 | 0 | 0 | 0 |
| TTTTTC | 0 | 0 | 0 | 0 | 1 | 0 |
