# Supplementary material for: First comparative analysis of complete chloroplast genomes among six Hedysarum (Fabaceae) species
Source: Front Plant Sci. 2023 Aug 18;14:1211247. doi: 10.3389/fpls.2023.1211247 (PMC10473476; doi:10.3389/fpls.2023.1211247)
Supplement: Supplementary file 5 [file Table_5.doc]

**Supplementary Table 5. Individual characteristics of 76 protein-coding genes**

| No | Genes | Nucleotid diversity (Pi value) | Gene length (bp), after alignment | Gene type |
| --- | --- | --- | --- | --- |
| 1 | *psbA* | 0.00621 | 1062 | Reverse |
| 2 | *matk* | 0.01473 | 1518 | Reverse |
| 3 | *rbcL* | 0.01401 | 1428 | Reverse |
| 4 | *atpB* | 0.01559 | 1479 | Forward |
| 5 | *atpE* | 0.0209 | 408 | Forward |
| 6 | *ndhC* | 0.01267 | 363 | Forward |
| 7 | *ndhK* | 0.01704 | 675 | Forward |
| 8 | *ndhJ* | 0.00797 | 477 | Forward |
| 9 | *rps4* | 0.02031 | 624 | Forward |
| 10 | *ycf3* | 0.00971 | 507 | Forward |
| 11 | *psaA* | 0.0083 | 2274 | Forward |
| 12 | *psaB* | 0.00877 | 2205 | Forward |
| 13 | *rps14* | 0.01804 | 303 | Forward |
| 14 | *psbZ* | 0.00459 | 189 | Reverse |
| 15 | *psbC* | 0.01045 | 1422 | Reverse |
| 16 | *psbD* | 0.00847 | 1062 | Reverse |
| 17 | *psbM* | 0.00571 | 105 | Forward |
| 18 | *petN* | 0.0037 | 90 | Reverse |
| 19 | *rpoB* | 0.01435 | 3219 | Forward |
| 20 | *rpoC1* | 0.01907 | 2100 | Forward |
| 21 | *rpoC2* | 0.01958 | 4162 | Forward |
| 22 | *rps2* | 0.01839 | 711 | Forward |
| 23 | *atpI* | 0.01013 | 744 | Forward |
| 24 | *atpH* | 0.01192 | 246 | Forward |
| 25 | *atpF* | 0.0988 | 2277 | Forward |
| 26 | *atpA* | 0.02284 | 1569 | Forward |
| 27 | *psbI* | 0.01381 | 111 | Reverse |
| 28 | *psbK* | 0.00753 | 186 | Reverse |
| 29 | *accD* | 0.10858 | 2627 | Forward |
| 30 | *psaI* | 0.01905 | 105 | Forward |
| 31 | *ycf4* | 0.02033 | 636 | Forward |
| 32 | *cemA* | 0.0171 | 690 | Forward |
| 33 | *petA* | 0.01246 | 963 | Forward |
| 34 | *psbJ* | 0.02114 | 165 | Reverse |
| 35 | *psbL* | 0.00769 | 162 | Reverse |
| 36 | *psbF* | 0.005 | 120 | Reverse |
| 37 | *psbE* | 0.00238 | 252 | Reverse |
| 38 | *petL* | 0.01458 | 96 | Forward |
| 39 | *petG* | 0.00994 | 114 | Forward |
| 40 | *psaJ* | 0.00938 | 135 | Forward |
| 41 | *rpl33* | 0.02952 | 201 | Forward |
| 42 | *rps18* | 0.03293 | 360 | Forward |
| 43 | *rpl20* | 0.03508 | 363 | Reverse |
| 44 | *rps12* | 0.0095 | 393 | Reverse |
| 45 | *rpl2* | 0.01881 | 828 | Reverse |
| 46 | *clpP* | 0.16015 | 585 | Reverse |
| 47 | *psbB* | 0.00939 | 1527 | Forward |
| 48 | *psbT* | 0.01728 | 108 | Forward |
| 49 | *psbN* | 0.00909 | 132 | Reverse |
| 50 | *psbH* | 0.00631 | 222 | Forward |
| 51 | *petB* | 0.00776 | 648 | Forward |
| 52 | *petD* | 0.00856 | 483 | Forward |
| 53 | *rpoA* | 0.02122 | 1062 | Reverse |
| 54 | *rps11* | 0.02122 | 1062 | Reverse |
| 55 | *rpl36* | 0.01871 | 114 | Reverse |
| 56 | *rps8* | 0.02473 | 372 | Reverse |
| 57 | *rpl14* | 0.01951 | 369 | Reverse |
| 58 | *rpl16* | 0.01843 | 369 | Reverse |
| 59 | *rps3* | 0.03825 | 672 | Reverse |
| 60 | *rps19* | 0.01697 | 279 | Reverse |
| 61 | *rpl23* | 0.02411 | 282 | Reverse |
| 62 | *ycf2* | 0.03225 | 4211 | Forward |
| 63 | *ndhB* | 0.01504 | 1485 | Reverse |
| 64 | *rps7* | 0.02778 | 468 | Reverse |
| 65 | *ycf1* | 0.05239 | 5709 | Forward |
| 66 | *rps15* | 0.01245 | 273 | Forward |
| 67 | *ndhH* | 0.00728 | 1182 | Forward |
| 68 | *ndhA* | 0.0102 | 1092 | Forward |
| 69 | *ndhI* | 0.00549 | 516 | Forward |
| 70 | *ndhG* | 0.00891 | 531 | Forward |
| 71 | *ndhE* | 0.00572 | 303 | Forward |
| 72 | *psaC* | 0.00136 | 246 | Forward |
| 73 | *ndhD* | 0.01232 | 1524 | Forward |
| 74 | *ccsA* | 0.02092 | 972 | Reverse |
| 75 | *rpl32* | 0.01743 | 186 | Reverse |
| 76 | *ndhF* | 0.01229 | 2250 | Forward |
